# Supplementary material for: Large protein organelles form a new iron sequestration system with high storage capacity
Source: eLife. 2019 Jul 8;8:e46070. doi: 10.7554/eLife.46070 (PMC6668986; doi:10.7554/eLife.46070)
Supplement: Supplementary file 1. — All identified ferritins (Ftn), bacterioferritins (Btf) and DNA-binding proteins from starved cells (Dps) found in IMEF operon strains. IMEF and encapsulin capsid protein IDs are shown as well. 97% of IMEF operon-containing strains do not encode Ftn, 93% do not encode Bfr and 92% do not encode either. However, 93% of IMEF operon-encoding strains encode Dps systems. This likely indicates that the encapsulin based IMEF system represents the major iron storage system in 92% of the listed strains. It also indicates that IMEF systems do likely not function as unusual Dps system given that the vast majority of strains encode standard Dps system. Blast searches were carried out using the NCBI Blastp server with the following sequences as queries: Ftn: OTY20392, Bfr: EEK74551, Dps: WP_039234032, IMEF: WP_039238473, Encapsulin: WP_039238471. [file elife-46070-supp1.docx]

Supplementary file 1. Table of ferritin-like proteins (Flps) identified in IMEF operon-containing Firmicutes. All identified ferritins (Ftn), bacterioferritins (Btf) and DNA-binding proteins from starved cells (Dps) found in IMEF operon strains. IMEF and encapsulin capsid protein IDs are shown as well. 97% of IMEF operon-containing strains do not encode Ftn, 93% do not encode Bfr and 92% do not encode either. However, 93% of IMEF operon-encoding strains encode Dps systems. This likely indicates that the encapsulin based IMEF system represents the major iron storage system in 92% of the listed strains. It also indicates that IMEF systems do likely not function as unusual Dps system given that the vast majority of strains encode standard Dps system. Blast searches were carried out using the NCBI Blastp server with the following sequences as queries: Ftn: OTY20392, Bfr: EEK74551, Dps: WP_039234032, IMEF: WP_039238473, Encapsulin: WP_039238471.

| **Organism** | **Ftn** | **Bfr** | **Dps** | **IMEF** | **Encapsulin** |
| --- | --- | --- | --- | --- | --- |
| [Bacillus] aminovorans DSM 1314 | no | no | OAH56278 | WP_063965031 | WP_063965030 |
| [Bacillus] aminovorans DSM 4337 | no | no | OAH62575 | OAH53583 | OAH53584 |
| Alteribacillus bidgolensis P4B | no | WP_091586138 | no | SDI49454 | SDI49426 |
| Alteribacillus iranensis DSM 23995 | no | WP_091663879 | WP_091663394 | WP_091658128 | WP_091658125 |
| Aneurinibacillus tyrosinisolvens | OIJ04211 | no | WP_071319460 | WP_047151354 | WP_047151353 |
| Aneurinibacillus migulanus DSM 2895 | WP_043066706 | WP_043065797 | WP_021619211 | WP_043068886 | WP_043068885 |
| Aneurinibacillus sp. XH2 | no | no | WP_057898153 | PGF_00166540 | WP_043068885 |
| Aneurinibacillus thermoaerophilus L 420-91 | no | no | WP_091260354 | SDH31038 | SDH31061 |
| Quasibacillus thermotolerans MTCC 8252 | no | no | WP_039234032 | WP_039238473 | WP_039238471 |
| Bacillus azotoformans LMG 9581 | no | no | WP_087946144 | EKN64196 | EKN64195 |
| Bacillus azotoformans MEV2011 | no | no | WP_035195831 | KEF38094 | KEF38093 |
| Bacillus methanolicus MGA3 | no | no | WP_003347246 | AIE58873 | AIE58874 |
| Bacillus methanolicus PB1 | no | no | WP_003351303 | WP_003351118 | WP_003351117 |
| Bacillus sp. 1NLA3E | no | no | WP_015595456 | WP_015593352 | WP_015593353 |
| Bacillus sp. FJAT-27238 | no | no | KMZ44901 | WP_057773495 | WP_057773497 |
| Bacillus sp. OK048 | no | no | WP_090761658 | WP_090761391 | WP_090761393 |
| Bacillus sp. OV166 | no | no | WP_088089380 | SMQ84107 | SMQ84105 |
| Bacillus sp. strain JF8 | no | no | AGT33197 | AGT31241 | AGT31240 |
| Bacillus thermotolerans SGZ-8 | no | no | WP_039234032 | QY97_0899 | WP_015593353 |
| Brevibacillus agri BAB-2500 | no | no | WP_081592058 | ELK43221 | ELK43220 |
| Brevibacillus borstelensis AK1 | no | no | WP_003388077 | WP_003389153 | WP_003389152 |
| Brevibacillus brevis NBRC 100599 | no | no | BAH42285 | WP_015892426 | WP_007721249 |
| Brevibacillus brevis ATCC 35690 | no | no | no | WP_016742089 | WP_007721249 |
| Brevibacillus brevis DZQ7 | no | no | WP_083261598 | WP_064202172 | WP_007721249 |
| Brevibacillus choshinensis DSM 8552 | no | no | WP_055747325 | WP_055744591 | WP_055744592 |
| Brevibacillus formosus DSM 9885 | no | no | WP_047070550 | WP_047071899 | WP_007721249 |
| Brevibacillus formosus NF2 | no | no | WP_088906278 | WP_047071899 | WP_007721249 |
| Brevibacillus laterosporus DSM 25 | no | no | WP_018671993 | WP_003334817 | WP_003334816 |
| Brevibacillus laterosporus GI-9 | no | no | WP_018671993 | WP_003334817 | WP_003334816 |
| Brevibacillus laterosporus LMG 15441 | no | no | WP_018671993 | WP_003334817 | WP_003334816 |
| Brevibacillus panacihumi W25 | no | no | WP_023556060 | WP_023557771 | WP_023557772 |
| Brevibacillus parabrevis CN1 | no | no | WP_083955677 | WP_122964006 | WP_063228109 |
| Brevibacillus reuszeri DSM 9887 | no | no | WP_103109718 | WP_049742502 | WP_049742503 |
| Brevibacillus sp. BC25 | no | no | WP_007726053 | WP_007721247 | WP_007721249 |
| Brevibacillus sp. CF112 | no | no | WP_007781621 | WP_007783174 | WP_007783173 |
| Brevibacillus sp. OK042 | no | no | WP_092266183 | WP_092268779 | WP_092268777 |
| Brevibacillus sp. SKDU10 | no | no | WP_082890860 | WP_064017301 | WP_003334816 |
| Brevibacillus sp. WF146 | no | no | WP_044898308 | WP_029098686 | WP_065067008 |
| Domibacillus antri | no | no | WP_075399286 | WP_075398991 | WP_075398990 |
| Domibacillus enclensis DSM 25145 | no | no | WP_045852489 | WP_045851598 | WP_045851597 |
| Domibacillus iocasae DSM 29979 | no | no | WP_069938694 | WP_069939583 | WP_069939582 |
| Geobacillus kaustophilus GBlys | no | no | WP_014196600 | WP_044731961 | WP_044731962 |
| Geobacillus kaustophilus Et2/3 | no | no | WP_044733039 | BAD75202 | BAD75201 |
| Geobacillus lituanicus N-3 | no | no | WP_033014567 | WP_047757677 | WP_100659874 |
| Geobacillus sp. 12AMOR1 | no | no | AKM20138 | AKM18226 | AKM18225 |
| Geobacillus sp. 15 | no | no | KZM53562 | KZM56073 | KZM56072 |
| Geobacillus sp. 46C-IIa | no | no | WP_081208050 | WP_081207017 | WP_081206634 |
| Geobacillus sp. A8 | no | no | WP_011232333 | WP_014195239 | WP_119877568 |
| Geobacillus sp. B4113_201601 | no | no | WP_033018292 | WP_033843367 | WP_119877568 |
| Geobacillus sp. CAMR5420 | no | no | KDE47147 | WP_033024921 | WP_033024920 |
| Geobacillus sp. LC300 | no | no | AKU26136 | AKU27458 | AKU27457 |
| Geobacillus sp. MAS1 | no | no | ESU73485 | ESU72279 | ESU72278 |
| Geobacillus sp. PA-3 | no | no | KQB92177 | KQB94129 | no |
| Geobacillus sp. Sah69 | no | no | KQC46690 | KQC48288 | KQC48287 |
| Geobacillus sp. Y412MC52 | no | no | ADU95335 | ADU93311 | ADU93310 |
| Geobacillus stearothermophilus 10 | no | no | ALA69799 | WP_013523228 | WP_013523227 |
| Geobacillus stearothermophilus strain 22 | no | no | OAO85981 | WP_049626374 | WP_049626373 |
| Geobacillus stearothermophilus strain 53 | no | no | WP_033014567 | WP_033024921 | WP_033024920 |
| Geobacillus stearothermophilus strain A1 | no | no | KMY59742 | KMY59381 | KMY59380 |
| Geobacillus stearothermophilus strain B4109 | no | no | WP_033014567 | WP_033024921 | WP_033024920 |
| Geobacillus stearothermophilus strain B4114 | no | no | WP_033014567 | WP_033024921 | WP_033024920 |
| Geobacillus subterraneus KCTC 3922 | no | no | WP_063164935 | AMX84296 | AMX84297 |
| Geobacillus thermocatenulatus BGSC 93A1 | no | no | WP_025950204 | WP_014195239 | WP_119877568 |
| Geobacillus thermodenitrificans T12 | no | no | WP_008880962 | EDY07466 |  |
| Geobacillus thermoleovorans B23 | no | no | WP_011232333 | WP_014195239 | WP_011230417 |
| Geobacillus thermoleovorans CCB_US3_UF5 | no | no | AGE23447 | AEV18405 | AEV18404 |
| Geobacillus thermoleovorans strain FJAT-2391 | no | no | AKU26136 | AWO73557 | AWO73558 |
| Lihuaxuella thermophila strain DSM 46701 | no | WP_089964625 | no | WP_089972673 | WP_089972676 |
| Sporosarcina globispora DSM 4 | no | no | WP_053434019 | WP_053433170 | WP_053437549 |
| Thalassobacillus cyri CCM7597 | no | WP_093043674 | no | WP_093041210 | WP_093041212 |
| Thermoflavimicrobium dichotomicum DSM 44778 | no | no | no | WP_093230746 | WP_093230618 |
|  |  |  |  |  |  |
| **Absent in:** | **97%** | **93%** | **7%** |  |  |
|  |  |  |  |  |  |
|  | **Ftn and Bfr missing: 92%** | |  |  |  |
