# Supplementary material for: Large protein organelles form a new iron sequestration system with high storage capacity
Source: eLife. 2019 Jul 8;8:e46070. doi: 10.7554/eLife.46070 (PMC6668986; doi:10.7554/eLife.46070)
Supplement: Supplementary file 2. [file elife-46070-supp2.docx]

Supplementary file 2. Table of cryo-EM data collection statistics for IMEF-loaded encapsulin.

| **Cryo-EM data collection and processing** | **IMEF encapsulin** |
| --- | --- |
| Electron microscope | Tecnai F20 |
| Voltage (kV) | 200 |
| Electron dose (e^-^/Å^2^) | 44 |
| Physical pixel size (Å) | 1.28 |
| Number of collected movies | 601 |
| Defocus range (avg) (μm) | 1.0-3.0 (2.3) |
| Particle number for final map | 18,995 |
| Symmetry for final map | I (icosahedral) |
| Resolution (Å) | 3.85 |
| Map sharpening B-factor (Å^2^) | -151 |
| **Atomic model refinement (ASU)** |  |
| Number of chains in ASU | 7 (4 Enc, 3 TP) |
| Number of protein residues | 1130 |
| Number of atoms | 9064 |
| **Geometric parameters (r.m.s.d.)** |  |
| Bond length (Å) | 0.007 |
| Bond angle (º) | 0.941 |
| **Ramachandran statistics** |  |
| Residues favoured (%) | 87.4 |
| Residues allowed (%) | 12.6 |
| Residues disallowed (%) | 0.0 |
| Rotamer outliers (%) | 0.61 |
| Clashscore | 3.96 |
