# Supplementary material for: Large protein organelles form a new iron sequestration system with high storage capacity
Source: eLife. 2019 Jul 8;8:e46070. doi: 10.7554/eLife.46070 (PMC6668986; doi:10.7554/eLife.46070)
Supplement: Supplementary file 3. — IMEF: Iron-mineralizing encapsulin-associated Firmicute cargo, EncFlp: ferritin-like proteins (Flps) found within encapsulin operons containing targeting peptides, noEncFlp: Flps found outside encapsulin operons not containing a targeting peptide, Bfr: bacterioferritin, Rr: rubrerythrin, Mam-Ftn: mammalian ferritin, Bac-Ftn: bacterial ferritin, Dps: DNA-binding proteins from starved cells. [file elife-46070-supp3.docx]

Supplementary file 3. List of ferritin-like protein and IMEF protein IDs used to construct the phylogenetic tree shown in Figure 3A. IMEF: Iron-mineralizing encapsulin-associated Firmicute cargo, EncFlp: ferritin-like proteins (Flps) found within encapsulin operons containing targeting peptides, noEncFlp: Flps found outside encapsulin operons not containing a targeting peptide, Bfr: bacterioferritin, Rr: rubrerythrin, Mam-Ftn: mammalian ferritin, Bac-Ftn: bacterial ferritin, Dps: DNA-binding proteins from starved cells.

| **Flp-type** | **Protein ID** |
| --- | --- |
| IMEF | WP_018672703 |
| IMEF | WP_005828111 |
| IMEF | WP_044897354 |
| IMEF | WP_003332271 |
| IMEF | WP_035178714 |
| IMEF | WP_028782218 |
| IMEF | WP_04180969 |
| IMEF | WP_031404939 |
| IMEF | WP_028401721 |
| IMEF | WP_003348284 |
| IMEF | WP_014195239 |
| IMEF | WP_045851598 |
| IMEF | WP_046179756 |
| IMEF | IMEF |
| IMEF | WP_045518068 |
| IMEF | WP_028401721 |
| IMEF | WP_003348284 |
| IMEF | WP_043068886 |
| IMEF | WP_047151354 |
| EncFlp | ACL70345 |
| EncFlp | CAE09437 |
| EncFlp | ABR50494 |
| EncFlp | TmFlp |
| EncFlp | CAN95442 |
| EncFlp | ACY16335 |
| EncFlp | CAJ63146 |
| EncFlp | RrFlp |
| noEncFlp | AJP49228 |
| noEncFlp | ACV25530 |
| noEncFlp | AHK79895 |
| noEncFlp | AEF99408 |
| noEncFlp | CCE22839 |
| noEncFlp | AFL74931 |
| noEncFlp | ACL71928 |
| noEncFlp | AAZ98418 |
| noEncFlp | AHF03132 |
| noEncFlp | AKH19188 |
| noEncFlp | AKH68756 |
| noEncFlp | AGS38469 |
| noEncFlp | AFZ34279 |
| noEncFlp | CAD84078 |
| noEncFlp | BAP56432 |
| noEncFlp | ACB49386 |
| EncFlp | ABF90650 |
| EncFlp | MxFlp |
| EncFlp | ADO71860 |
| EncFlp | ABF92698 |
| EncFlp | ABN52731 |
| EncFlp | ABS55612 |
| EncFlp | ABV34473 |
| EncFlp | AAL81316 |
| EncFlp | AFN03982 |
| Bfr | EAQ77221 |
| Bfr | BAF71400 |
| Bfr | CAJ12841 |
| Bfr | ABQ95053 |
| Bfr | ABR71746 |
| Bfr | ABC22995 |
| Bfr | BFR_ECOLI |
| Bfr | AKH68832 |
| Bfr | CEL31094 |
| Bfr | BFR_AZOVI |
| Bfr | KIL04563 |
| Bfr | EPP19171 |
| Bfr | KMN33871 |
| Bfr | AJQ92466 |
| Bfr | CUR48233 |
| Bfr | ABC24268 |
| Bfr | ABR86521 |
| Rr | AAB88944 |
| Rr | AAB85322 |
| Rr | ACM24105 |
| Rr | CEP79124 |
| Rr | AIG98643 |
| Rr | AEH24665 |
| Rr | Q9V0A0_PYRAB |
| Rr | KUH34502 |
| Rr | Q5JF11_PYRKO |
| Rr | 3MPS_A |
| Rr | AAB90407 |
| Rr | AAB90419 |
| Rr | RUBY_DESVH |
| Rr | EEG74317 |
| Rr | 1J30_A |
| Rr | 4DI0_A |
| Rr | EDL54893 |
| Rr | AAK86067 |
| Mam-Ftn | XP_018870100 |
| Mam-Ftn | FRIL_PONAB |
| Mam-Ftn | FRIL_HUMAN |
| Mam-Ftn | FRIL1_MOUSE |
| Mam-Ftn | NP_001266795 |
| Mam-Ftn | XP_005005114 |
| Mam-Ftn | BAG82928 |
| Mam-Ftn | ELR56618 |
| Mam-Ftn | AAH61303 |
| Mam-Ftn | FRI2_LITCT |
| Mam-Ftn | FRIM_SALSA |
| Mam-Ftn | FRIH_TRENE |
| Mam-Ftn | FTMT_HUMAN |
| Mam-Ftn | 2FHA |
| Mam-Ftn | FRIH_MOUSE |
| Bac-Ftn | 4ITW_A |
| Bac-Ftn | EAQ74833 |
| Bac-Ftn | BFRB_MYCTE |
| Bac-Ftn | ABQ03725 |
| Bac-Ftn | EDM65666 |
| Bac-Ftn | FTNA_ECOLI |
| Bac-Ftn | CAL34758 |
| Bac-Ftn | FTN_HELPJ |
| Bac-Ftn | ABN51258 |
| Bac-Ftn | 2JD7_A |
| Bac-Ftn | ABT93906 |
| Bac-Ftn | ADD56915 |
| Dps | DPS_CAMJE |
| Dps | ABC21138 |
| Dps | AEP02251 |
| Dps | KIO64845 |
| Dps | DPS_BREBE |
| Dps | KKX55325 |
| Dps | PCN43109 |
| Dps | KPC73845 |
| Dps | AHX18156 |
| Dps | KQL50044 |
| Dps | ACS25476 |
| Dps | OQP01613 |
| Dps | EQB94255 |
| Dps | WP_044733039 |
| Dps | PAY11715 |
| Dps | Q65FU7_BACLD |
| Dps | DP_STRSU |
| Dps | DPS_LISMO |
| Dps | EAQ79922 |
| Dps | DPS_MYCSM |
| Dps | DPS_AGRFC |
| Dps | DPS_ECOLI |
| Dps | ABC22300 |
