# Supplementary material for: Large protein organelles form a new iron sequestration system with high storage capacity
Source: eLife. 2019 Jul 8;8:e46070. doi: 10.7554/eLife.46070 (PMC6668986; doi:10.7554/eLife.46070)
Supplement: Supplementary file 4. [file elife-46070-supp4.docx]

Supplementary figure 4. X-ray structure determination and refinement statistics for the IMEF cargo protein.

| PDB ID | 6N63 |
| --- | --- |
| Unit cell (Å)*^a^* | a = b = 81.4, c = 65.9 |
| Spacegroup | P 4_1_ 2_1_ 2 |
| Resolution range (Å)*^a^* | 58.0 – 1.72 (1.78 – 1.72) |
| Wavelength (Å) | 0.9762 |
| Observed reflections | 219,893 |
| Unique reflections | 24,131 |
| Completeness (%) | 99.7 (100.0) |
| Redundancy | 9.1 (8.1) |
| R_pim_(%)*^b^* | 0.026 (0.504) |
| Overall <*I*/σ(*I*)> | 29.0 (1.0) |
| CC_1/2_ | 0.982 (0.681) |
| *R*_cryst_*^c^/R*_free_*^c^* (%) | 19.4/22.0 |
| Ramachandran plot (%)  Favored/allowed/outliers | 97.8 / 2.2 / 0.0 |
| Bond lengths*^d^* (Å) | 0.018 |
| Bond angles*^d^* (°) | 1.430 |
| Average B-factors (Å)  Protein  Waters  Other | 49.4  52.3  40.5 |

*a*Values in parentheses are for the highest resolution shell.

*^b^*$R_{pim}=\sqrt{\frac{1}{n-1}}\frac{\sum\left| I-\left\langle I \right\rangle\right|}{\sum I}$

Where *I* is the observed integrated intensity, <*I*> is the average integrated intensity obtained from multiple measurements, and the summation is over all observed reflections.

*^c^*$R_{cryst}=\frac{\sum\left| \left| F_{o} \right|-k\left| F_{c} \right| \right|}{\sum F_{o}}$

*F*_o_ and *F*_c_ are the observed and calculated structure factors, respectively, and *k* is a scaling factor. The summation is over all measurements. *dR*_free_ is calculated as *R*_cryst_ using 5% of the reflections chosen randomly and omitted from the refinement calculations. Model stereochemistry was analyzed using MolProbity (*49*).

*e*Bond lengths and angles are root-mean-square deviations from ideal values.
