# Supplementary material for: Large protein organelles form a new iron sequestration system with high storage capacity
Source: eLife. 2019 Jul 8;8:e46070. doi: 10.7554/eLife.46070 (PMC6668986; doi:10.7554/eLife.46070)
Supplement: Supplementary file 5. — #Fe from EELS analysis values were extracted using Gatan software. Errors combine estimated statistical error in this measurement with known error for cross section. For density calculations, particles were approximate as spheres. [file elife-46070-supp5.docx]

Supplementary figure 5. EELS data of electron dense cores of purified IMEF encapsulins produced in *E. coli* under high iron conditions. #Fe from EELS analysis values were extracted using Gatan software. Errors combine estimated statistical error in this measurement with known error for cross section. For density calculations, particles were approximate as spheres.

| **Particle ID** | **Diameter (nm)** | **Diameter Standard deviation (nm)** | **# Fe atoms from EELS analysis** | **Error in # Fe atoms** | **Calculated density (# Fe atoms/nm^3^)** |
| --- | --- | --- | --- | --- | --- |
| 1 | 22.8 | 1.45 | 19070 | 3756.79 | 3.07 |
| 2 | 20.5 | 1.37 | 7539 | 1485.18 | 1.67 |
| 3 | 19.2 | 2.38 | 8286 | 1632.34 | 2.25 |
| 4 | 12 | 1.73 | 2512 | 494.86 | 2.78 |
| 5 | 16.9 | 2.57 | 2836 | 558.69 | 1.13 |
| 6 | 19.4 | 3.64 | 8171 | 1609.69 | 2.13 |
| 7 | 15.4 | 0.46 | 3137 | 617.99 | 1.64 |
| 8 | 20.8 | 0.64 | 7327 | 1443.42 | 1.55 |
| 9 | 20.7 | 0.78 | 6574 | 1295.08 | 1.41 |
| 10 | 16.5 | 1.95 | 3936 | 775.39 | 1.66 |
| 11 | 19.8 | 4.43 | 7250 | 1428.25 | 1.78 |
| 12 | 20.3 | 2.70 | 9526 | 1876.62 | 2.17 |
| 13 | 23.6 | 1.80 | 23293 | 4588.72 | 3.40 |
| 14 | 19.5 | 2.36 | 7785 | 1533.65 | 2.00 |
| 15 | 18.7 | 2.40 | 3425 | 674.725 | 1.01 |
| 16 | 21.1 | 1.29 | 6721 | 1324.04 | 1.38 |
| 17 | 20.5 | 1.98 | 10170 | 2003.49 | 2.24 |
| 18 | 21.4 | 3.68 | 9840 | 1938.48 | 1.91 |
| 19 | 24.7 | 2.74 | 10968 | 2160.70 | 1.39 |
| 20 | 25.1 | 2.35 | 14739 | 2903.58 | 1.77 |
| 21 | 28.6 | 2.19 | 13124 | 2585.43 | 1.07 |
| 22 | 22.23 | 1.37 | 6783 | 1336.25 | 1.17 |
| Average |  |  |  |  | 1.85 |
